
JOURNAL INFORMATION
==============================
NLM Title Abbreviation: ScientificWorldJournal
Journal ID: ScientificWorldJournal
NLM Journal ID: 101131163
Journal ID: TSWJ

The Scientific World Journal

ISSN: 2356-6140
EISSN: 1537-744X
Publisher: Wiley

ARTICLE INFORMATION
==============================
PMCID: PMC6084256
DOI: 10.1100/tsw.2001.445
Article ID: 831961
Article version: 1
Subjects: Extended Abstract

Immunohistochemical Localization of Calcitonin Receptor-Like Receptor and Receptor Activity Modifying Proteins in Human Cerebral and Cranial Vasculature

Wainwright A. 1
Edvinsson L. 2
Oliver K. R. 1
1 Merck Research Laboratories, Neuroscience Research Centre, Harlow, Essex CM20 2QR, UK
2 Department of Internal Medicine, University Hospital, 221 85 Lund, Sweden
Publication date: 2001
Electronic publication date: 2001 Nov 27
Volume: 1
First page: 48
Last page: 48
Copyright: Copyright © 2001 A. Wainwright et al.
Copyright year: 2001
License: This is an open access article distributed under the Creative Commons Attribution License, which permits unrestricted use, distribution, and reproduction in any medium, provided the original work is properly cited.
License URL: https://creativecommons.org/licenses/by/3.0/

==============================
4th International Meeting on Calcitonin Gene-Related Peptide (CGRP)
*TheScientificWorld* (2001) 1(S1), 48
ISSN 1532-2246; DOI 10.1100/tsw.2001.445

### IMMUNOHISTOCHEMICAL LOCALIZATION OF CALCITONIN RECEPTOR-LIKE RECEPTOR AND RECEPTOR ACTIVITY MODIFYING PROTEINS IN HUMAN CEREBRAL AND CRANIAL VASCULATURE

A. Wainwright[1], L. Edvinsson[2], and K.R. Oliver[1]
[1]Merck Research Laboratories, Neuroscience Research Centre, Harlow, Essex CM20 2QR, U.K.;
[2]Department of Internal Medicine, University Hospital, 221 85 Lund, Sweden

Calcitonin gene-related peptide and adrenomedullin are both members of a structurally related neuropeptide family. Both are potent vasodilators and known to be expressed in the trigeminovascular system. Central to functional binding of these neuropeptides is calcitonin receptor-like receptor (CRLR), a G-protein coupled receptor whose cell surface expression and pharmacology is determined by co-expression of a receptor activity-modifying protein (RAMP). CRLR combined with RAMP1 binds CGRP with high affinity, whilst CRLR coexpression with either RAMP2 or RAMP3 confers high affinity binding of adrenomedullin. We sought to investigate expression of these receptor components in human cerebral and cranial vasculature to further characterize CGRP receptor family content and potential function(s) in this vascular system. Antisera specific for CRLR, RAMP1, RAMP2, and RAMP3 were raised, characterized, and applied to tissue sections utilizing modern immunohistochemical techniques and confocal microscopy. Localization data demonstrated for the first time, the presence of these receptor component proteins in human cerebrovasculature, and suggest that both CGRP and adrenomedullin receptors may arise from coassembly of these accessory proteins with CRLR. These novel data advance our understanding of the function of the trigeminovascular system and the normal and potential pathophysiological role of CGRP family neuropeptides.
